# Comparing Bariatric Surgery Patients Who Desire, Have Undergone, or Have No Desire for Body Contouring Surgery: a 5-Year Prospective Study of Body Image and Mental Health

**DOI:** 10.1007/s11695-022-06117-6

**Published:** 2022-06-23

**Authors:** Liliana Buer, Ingela Lundin Kvalem, Silje Bårdstu, Tom Mala

**Affiliations:** 1 Department of Psychology, University of Oslo, PB 1094, Blindern, N-0317 Oslo, Norway; 2 Norwegian Institute of Public Health, PB 222, 0213 Skøyen, Oslo Norway; 3 Department of Gastrointestinal and Pediatric Surgery, and Department of Endocrinology, Morbid Obesity and Preventive Medicine, Oslo University Hospital, 4950 0424 Oslo, PB Norway; 4 Institute of Clinical Medicine, University of Oslo, Oslo, Norway

**Keywords:** Bariatric surgery, Body contouring surgery, Body image, Mental health

## Abstract

**Purpose:**

After bariatric surgery, body contouring surgery (BCS) is thought to improve body image, weight loss, and mental health. Many patients desire but do not undergo BCS after bariatric surgery. This patient subset has rarely been studied. The present study compares bariatric surgery patients that, at 5 years after surgery, desires, have undergone or have no desire for BCS regarding pre- and post-surgery body image and mental health, including within-group changes over time.

**Materials and Methods:**

Data were collected from participants (*N* = 216) pre-bariatric surgery and at 1- and 5-year post-surgery. Health care providers measured body mass index (BMI). All other data were collected via self-report (questionnaires).

**Results:**

At 5-year post-surgery, 30.6% had undergone BCS, 17.1% did not desire it, and 52.3% desired BCS. Patients who subsequently desired BCS scored lower on body satisfaction pre-surgery than the other groups. They also reported less resilience pre-surgery and more depressive symptoms at all times compared to participants with BCS. For five-year post-surgery, patients who desired BCS had lower body satisfaction levels than patients with BCS and were more bothered with excess skin relative to the two other groups. Body satisfaction improved in all three groups from baseline to five years and in most patients with BCS. Mental health improved only in patients with BCS.

**Conclusion:**

This study emphasizes the relevance of identifying participants who desire but have not undergone BCS. The study suggests that BCS is associated with improved body image and mental health.

**Graphical abstract:**

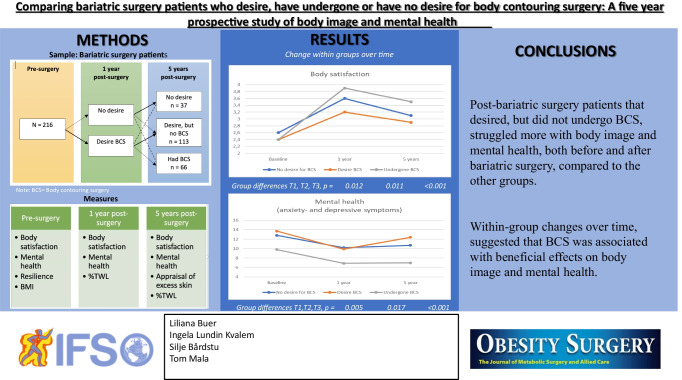

## Introduction

Despite the benefits of bariatric surgery in terms of psychosocial well-being, variations in long-term effects across individual patients and patient groups are not well understood [1]. Most studies suggest long-term positive consequences [1], but psychosocial improvements may deteriorate after some years [2, 3]. Following substantial weight loss after surgery, more than 90% report excessive skin [4, 5], which may negatively influence physical, social, and psychological functioning [6], including body image [7–9]. About 70–90% desire body contouring surgery (BCS) after bariatric surgery [10, 11], although only 15–30% undergo BCS [12, 13]. The large group of patients who desire but do not undergo BCS in this context has rarely been investigated separately and never in any prospective study.

Among bariatric surgery patients, there is a high prevalence of body dissatisfaction and symptoms of mood disorders compared to the general population [14, 15]. Undergoing BCS is associated with better maintenance of weight loss, fewer challenges related to excess skin [16, 17], and improved body satisfaction [1, 9]. Regarding mental health, some studies have found positive effects of BCS [1, 12, 18], while others have found small [19] or no effects [7]. A cross-sectional study that specifically examined patients who desired but had not undergone BCS found more body dissatisfaction and depressive symptoms in this group than those with BCS and no desire for BCS [20].

Factors known to be protective against symptoms of mood disorders and maladjustment (i.e., resilience) [21, 22] are, as far as we know, unexplored in the contexts of bariatric surgery. Prospective studies regarding psychological functioning before bariatric surgery and how this interact with desiring, not desiring, and undergoing BCS after bariatric surgery are sparse.

The main aim of this study was to explore potential differences between participants that have undergone BCS, those that desire without having undergone BCS, and those that have no desire for BCS after bariatric surgery. Group differences regarding body image and mental health at baseline, 1 year, and 5 years after bariatric surgery were examined, including changes in body satisfaction and mental health over time. Since variation in weight and weight loss may explain group differences in body image and mental health [20, 23–25], these variables were controlled for in the analysis.

## Methods

### Participants and Procedures

The data were retrieved from the Oslo Bariatric Surgery Study, a prospective cohort study [26]. Participants were recruited at Oslo University Hospital between 2011 and 2013. Inclusion criteria for study participation were *BMI* ≥ 40 or *BMI* ≥ 35 kg/m^2^ with obesity-related comorbidity, age ≥ 18 years, and the ability to understand and comply with the study procedures. During the pre-surgical consultation, the surgeons administered information sheets and consent forms. All questionnaires were mailed to the Department of Psychology. Out of 506 eligible patients, 332 agreed to participate, and 302 completed the baseline questionnaire. A total of 16 participants withdrew their consent or withdrew from the surgery. Out of the 316 participants, 257 (81%) returned the questionnaire one year post-surgery, and 222 out of 302 (74%) returned the questionnaire five years post-surgery.

This study included 216 participants with complete data at the baseline and 5-year post-surgery. A total of 16 participants underwent sleeve gastrectomy, and 200 (92.6%) underwent Roux-en-Y gastric bypass. Attrition from the study was analyzed using multiple logistic regression analysis. Additionally, the study variables at baseline were included as predictors of non-participation five years later. Participants who were not employed (*OR* = 2.20; 95% *CI* = 1.20–4.03, *p* = 0.011) at baseline were more likely to have dropped out of the study.

### Measures

For five-year post-surgery, participants were asked whether they had undergone BCS, and those who confirmed were labeled “BCS.” Participants without BCS were asked, “How much do you desire to have plastic surgery?” with five response options: “not at all,” “not sure,” “a bit,” “quite a lot,” and “very much.” Participants who answered “not at all” were labeled as “no desire” (ND), while those that answered “a bit,” “quite a lot,” and “very much” were labeled as “desire” (D). Participants were also asked if they desired BCS (yes/no) 1-year post-surgery. Those who had answered “yes” at 1 year and were “unsure” at 5 years were placed in the group “desire,” and those who did not desire BCS at 1 year and were “unsure” at 5 years were placed in the “ND” group.

*Body image* was measured using subscales from the Multidimensional Body Self Relations Questionnaire [27]: The Body Area Satisfaction Scale (BASS; 9 items) and Appearance Orientation (AO; 14 items, only baseline). Both scales had response categories ranging from 1 to 5*.* High mean scores on the BASS indicated satisfaction with body areas, weight, and general appearance, and on AO high scores signified preoccupation with appearance and body*.*

*Appraisal of excess skin* was measured 5-year post-surgery on the 7-item subscale Body-Q Appearance [28], with response options ranging from 1 = “extremely bothered” to 4 = “not at all bothered.”

*Mental health* was measured using the Hospital Anxiety and Depression Scale (HADS) [29], with response options ranging from 0 to 3. Scores were added to yield sum scores. Total scores above 15 indicates a possible need for treatment. The two subscales regarding depressive and anxiety symptoms (seven items each), reflect mental health. For each subscale, scores between 8 and 10 indicate mild symptoms, scores between 11 and 14 indicate moderate symptoms, and scores between 15 and 21 indicate severe symptoms [30].

*Resilience* was measured at baseline using the Resilience Scale for Adults (33 items with five response options for each [31]. The scale reflects factors important for preventing maladjustment and mental health problems, such as personal strength, social competence, family cohesion, social resources, and structural style. High mean scores indicate greater resilience.

*Body mass index*, BMI (kg/m^2^), was measured using a platform scale (Seca 635, III; 0–300 kg). Weight loss was calculated as the percent total weight-loss (%TWL).

### Statistical Analysis

Mean group differences were tested using one-way analysis of variance (ANOVA) and Bonferroni post hoc tests. Repeated measures of variance were used for changes over time within the groups. Analysis of covariance (ANCOVA) was used to adjust for possible confounders. To include BMI/%TWL as a covariate, the following criteria had to be present: Groups differed on BMI/%TWL and the relevant dependant variable (e.g., body satisfaction). Furthermore, BMI/%TWL had to predict variance in the dependent variable.

## Results

The sample comprised 76.9% women (*n* = 166). Pre-surgery mean age was 44.6 years (*SD* = 9.20), and the mean BMI was 43.9 kg/m^2^ (*SD* = 5.46). One-year post-surgery mean %TWL was 29.4% (*SD* = 7.84), and at 5 years, 22.9% (*SD* = 9.83). At five-year post-surgery, 30.6% had undergone BCS (the BCS group), 52.3% desired BCS (the D group), and 17.1% did not desire BCS (the ND group) (Table 1). Of the 162 participants who desired BCS 1-year post-surgery, 35.2% had undergone BCS five years post-surgery, 61.1% still desired to do so, and 3.7% no longer desired BCS (Table 1).

**Table 1:** Distribution of demographics and desire for body contouring surgery (BCS) across the three patient groups: patients with no desire for BCS (ND), patients with a desire, but without having undergone BCS (D), and patients that had undergone BCS (BCS)

Five years after bariatric surgery	ND (*n* = 37)		D (*n* = 113)		BCS (*n* = 66)			
Gender	*n*	%	*n*	%	*n*	%	Chi-sq	*p*
Women	19	51.4	87	77	60	90.9	20.86	< 0.001
Men	18	48.6	26	23	6	9.1		
Total	37	100	113	100	66	100		
Employed (vs. not)	26	81.3	71	68.9	48	80.0	3.39	0.184
Have a partner/married (vs. not)	25	67.6	77	68.1	45	69.2	0.04	0.982
One year post-surgery								
Desire BCS	6	18.2	99	91.7	57	98.3	105.57	< 0.001
No desire for BCS	27	81.8	9	8.3	1	1.7		
Total	33	100	108	100	58	100		

*Chi-sq.* chi square (effect size)

The type of bariatric surgery did not differ between groups. The ND group included 48.6% males, compared to 23% and 9.1% among D and BCS participants, respectively (*p* ≤ 0.001). There were no group differences in age, relationship, or employment status. Pre-surgery mean BMI was higher in the D group (45.8 kg/m^2^) compared to the ND and BCS group (*p* = 0.002). One-year post-surgery average %TWL was higher in the BCS group than in the other groups (*p* = 0.001). At five-year post-surgery, the BCS group had 28.8%TWL, the D group had 21.6%, and the ND group had lost 18.3% since baseline (*p* ≤ 0.001).

At baseline, the ND group scored higher on body satisfaction than the D and BCS group (*p* = 0.012). Adjusted for variation in BMI, group differences were still significant. The groups did not differ in appearance orientation. The D group scored higher on depressive (*p* = 0.010) and anxiety symptoms (*p* = 0.043) and had lower levels of resilience than the BCS group (*p* = 0.007). Subscale scores for resilience showed significant differences for “family cohesion” and “social resources.” The ND group did not differ significantly from the other groups regarding mental health (Table 2).

**Table 2:** Differences in study variables pre-surgery, one year, and five years post Roux en Y bypass (*N* = 200) or gastric sleeve (*N* = 16) surgery across body contouring surgery (BCS) groups (ANOVA/ANCOVA)

	*ND* = no desire for BCS (*N* = 37)		*D* = desire BCS (*N* = 113)		*BCS* = undergone BCS (*N* = 66)						
Pre-surgery	*N*	Mean (SD)	*N*	Mean (SD)	*N*	Mean (SD)	*F*	*F adj*	*p*-value (adj. *p*-val.)	*η*^2^	Post hoc difference
Age	33	46.7 (9.58)	106	44.9 (9.49)	64	42.2 (8.52)	3.05		0.050	0.029	
BMI	33	42.9 (5.54)	106	45.8 (5.98)	64	43.0 (4.86)	6.31		0.002	0.059	D > ND; BCS
Body satisfaction	33	2.71 (0.61)	106	2.40 (0.56)	64	2.42 (0.48)	4.49	3.71	0.012 (0.026)	0.044	ND > BCS; D
Appearance orientation	33	3.16 (0.80)	106	3.46 (0.76)	64	3.55 (0.71)	3.03		0.051	0.029	
Depressive symptoms	32	5.53 (3.53)	105	6.05 (3.90)	63	4.29 (3.16)	4.67		0.010	0.045	D > BCS
Anxiety symptoms	32	6.81 (4.06)	105	7.67 (4.71)	63	5.94 (3.70)	3.20		0.043	0.031	D > BCS
Resilience	33	3.50 (0.69)	106	3.30 (0.83)	64	3.83 (0.55)	5.09		0.007	0.048	BCS > D
Personal strength	33	3.74 (0.76)	106	3.40 (0.79)	64	3.65 (0.63)	3.75		0.025	0.036	
Social competence	33	3.68 (0.68)	106	3.59 (0.87)	64	3.81 (0.81)	1.49		0.228	0.015	
Family cohesion	33	3.66 (0.90)	106	3.41 (1.01)	64	3.81 (0.94)	3.36		0.037	0.037	BCS > D
Social resources	33	4.15 (0.60)	106	3.94 (0.53)	64	4.30 (0.75)	5.02		0.007	0.048	BCS > D
Structural style	33	3.50 (0.69)	106	3.30 (0.83)	64	3.55 (0.77)	2.11		0.124	0.021	
One-year post-surgery	*N*	Mean	*N*	Mean	*N*	Mean	*F*	*F* adj	*p*-val. (*p*-val. adj.)	*η*^2^	Post hoc difference
%TWL	29	27.5 (8.38)	86	27.9 (7.48)	58	32.7 (7.67)	7.67		< 0.001	0.083	BCS > D; ND
Body satisfaction	34	3.59 (0.60)	108	3.20 (0.69)	59	3.32 (0.60)	4.59	5.15	0.011 (0.007)	0.044	ND > D
Depressive symptoms	34	3.50 (3.64)	107	3.54 (3.29)	63	2.05 (2.89)	4.28	2.77	0.015	0.042	D > BCS
Anxiety symptoms	34	6.38 (3.68)	106	6.40 (4.30)	60	4.93 (3.33)	2.79		0.064	0.028	
Five-year post-surgery	*N*	Mean	*N*	Mean	*N*	Mean	*F*	*F* adj	*p*-val. (*p* adj)	*η*^2^	Post hoc difference
%TWL	27	18.3 (9.69)	75	21.6 (9.83)	51	28.8 (9.98)	12.60		< 0.001	0.144	BCS > ND; D
Body satisfaction	37	3.16 (0.64)	112	2.93 (0.67)	66	3.47 (0.68)	14.04	21.27	< 0.001 (< 0.001)	0.117	BCS > D
Appraisal excess skin	37	3.22 (0.86)	112	2.06 (0.83)	63	2.76 (0.89)	30.92	30.27	< 0.001	0.228	ND > BCS > D
Depressive symptoms	37	4.05 (3.70)	111	4.63 (3.87)	65	2.32 (2.42)	9.19	3.85	< 0.001 (0.023)	0.080	D; ND > BCS
Anxiety symptoms	32	6.81 (4.06)	105	7.67 (4.71)	64	5.42 (3.98)	2.96		0.054	0.028	

*BMI*, body mass index; *%TWL*, percent total weight loss; *SD*, standard deviation; *η*.^2^, eta squared (effect size: small: 0.01; medium: 0.059; large: 0.138); *p adj*, adjusted for BMI/%TWL; post hoc difference: > “significantly higher scores than”

One-year post-surgery the ND group scored higher on body satisfaction, but only compared to the D group (*p* = 0.011). The difference remained when adjusted for %TWL. The D group reported more depressive symptoms than the BCS group (*p* = 0.015) concerning mental health.

At five-year post-surgery, the D group had lower body satisfaction than the BCS group (*p* ≤ 0.001), including when adjusted for %TWL. The ND group was less bothered with excess skin than the other two groups (*p* ≤ 0.001), and the BCS group was less bothered than the D group. The D group displayed more depressive symptoms (*p* ≤ 0.001) than the BCS and ND groups. Group differences in depressive symptoms remained when adjusted for %TWL (Table 2).

### Within-Group Changes in Body Satisfaction and Mental Health

All three groups showed improved body satisfaction from baseline to 1- and 5-year post-surgery (Table 3). From 1 to 5 years, satisfaction in the ND and D groups decreased, while scores for the BCS group remained stable. Only the BCS group’s mental health improved from baseline to 5 years. The D group improved from baseline to 1 year but had increased symptoms from one to 5 years. In the ND group, mental health did not change significantly.

**Table 3:** Changes in body satisfaction and mental health, as defined by the Body Area Satisfaction Scale scores and the Hospital Anxiety and Depressions Scale scores, between baseline (T1), one (T2), and 5 years (T3) after bariatric surgery within body contouring surgery (BCS) groups. Repeated measures ANOVA

Body satisfaction	ND (*N* = 30)	D (*N* = 102)	BCS (*N* = 59)
Sig. Wilks Lamba	< .001*	< .001*	< .001*
Eta square	.670	.637	.795
T1–T2	2.64–3.63*	2.39–3.18*	2.43–3.32*
T2–T3	3.63–3.17*	3.18–2.91*	3.32–3.47
T1–T3	2.64–3.17*	2.39–2.91*	2.43–3.47*
Mental health	ND (*N* = 28)	D (*N* = 94)	BCS (*N* = 56)
Sig. Wilks Lamba	.105	< .001 *	.002*
Eta square	.159	.251	.210
T1–T2	12.75–10.21	13.68–9.91*	9.82–6.91*
T2–T3	10.21–10.79	9.91–12.14*	6.91–7.04
T1–T3	12.75–10.79	13.68–12.14	9.82–7.04*

*BCS*, have undergone body contouring surgery; *ND*, no desire for BCS; *D*, desire for BCS; *Sig. Wilks Lamba and Eta square*, main effects; *significant change; Sig.Wilks Lamba: sig. value; < 0.05 significant change; *Eta square*, effect size of change

## Discussion

This study emphasizes differentiating between participants who have undergone, desire, or have no desire for BCS after bariatric surgery. The study revealed substantial differences across the three groups: After bariatric surgery, participants who desired, without having undergone BCS, had more body dissatisfaction and depressive symptoms and were more bothered with excess skin than to the other groups. The prospective design enabled an investigation of preoperative differences, not previously explored comparing these groups. The D group had already, at baseline, poorer mental health, and lower levels of resilience relative to patients who subsequently underwent BCS, and more body dissatisfaction compared to the group with no desire for BCS. For all groups, body satisfaction improved from baseline to 5 years, while mental health only improved among BCS-participants. One year after surgery, 81.4% of the participants desired BCS, comparable to previous findings [5]. Research suggesting that the desire for BCS declines with time [32, 33] was not supported. Four years later, only six participants had changed their mind from desiring to not desiring BCS. At 5 years, 17.1% had no desire for BCS, while 52.3% desired but had not undergone BCS, and 30.6% had undergone BCS. Consistent with previous research [20, 32], the BCS group included more women than the group that did not desire BCS. They also had the lowest BMI at baseline and the highest %TWL post-surgery.

Group differences in body satisfaction were observed at all times. At baseline, the ND group scored higher than the other groups, indicating that those who subsequently desired BCS, independent of whether they underwent it, had lower body satisfaction before surgery. We found no group differences in appearance orientation, supporting claims that investment in appearance might be unrelated to satisfaction [34, 35]. One year after surgery, the ND group still displayed the highest levels of body satisfaction, but only compared to those who desired BCS. At 5 years, body satisfaction and concerns about excess skin varied across groups in favor of participants with BCS relative to those desiring BCS. Post-surgery findings support previous research comparing groups with and without BCS [1, 17]. This study further elaborates on this by indicating that desire for BCS matters: Participants with no desire did not differ, neither at 1 or 5 years, from the BCS group in terms of body satisfaction, and were, notably less concerned about excess skin compared to both the D group and the BCS group who had removed some excess skin. This indicates that being bothered with excess skin is likely associated with a desire for BCS. Another study that explored the effects of desiring BCS without having it [9, 20] also found that those who desired BCS had the lowest levels of body satisfaction and reported the highest number of body parts affected by excess skin [20].

**Fig. 1:**
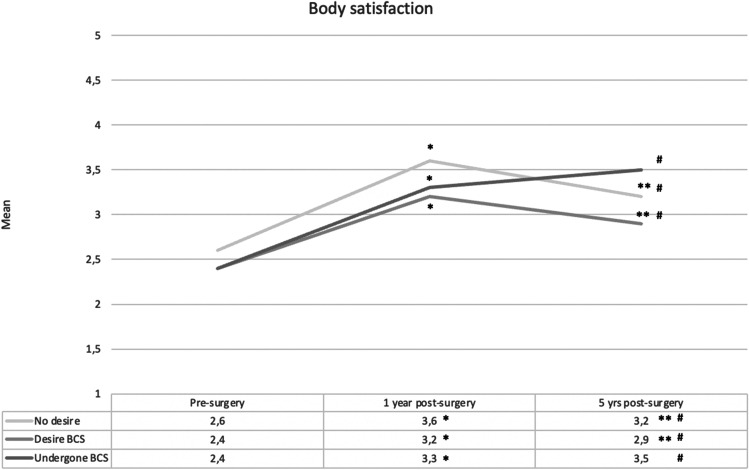
Changes in body satisfaction, as defined by the Body Area Satisfaction Scale scores, between baseline (T1), one (T2), and 5 years (T3) after bariatric surgery within body contouring surgery (BCS) groups. Note: *significant difference between T1 and T2; **significant difference between T2 and T3; #significant difference between T1 and T3

**Fig. 2:**
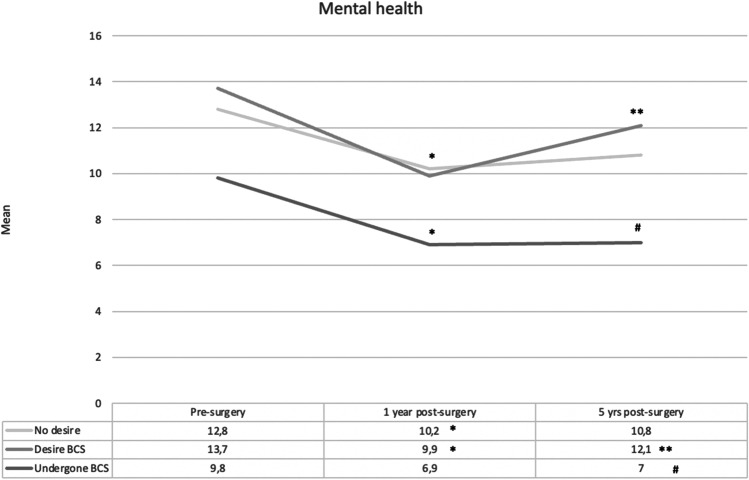
Changes in mental health, as defined by the Hospital Anxiety and Depression Scale scores, between baseline (T1), one (T2), and 5 years (T3) after bariatric surgery within body contouring surgery (BCS) groups. Note: *significant difference between T1 and T2; **significant difference between T2 and T3; #significant difference between T1 and T3

Mental health differed between groups at all time-points. Pre-surgery resilience scores showed that participants who subsequently desired but did not undergo BCS had more negative thoughts about themselves. Furthermore, they had more negative thoughts regarding coping with difficult situations (perception of self) and felt less close to and supported by friends/family (social resources and family cohesion) than BCS participants. Resilience factors are thought to be protective against mental health problems. The study supports this assumption, as the D group had more anxiety and depressive symptoms at baseline compared to BCS participants. After surgery, the same tendency was evident, with less depressive symptoms among BCS patients compared to those desiring BCS, at both 1 and 5 years, and compared to the ND group 5-year post-surgery. More depressive symptoms after surgery among patients desiring BCS were in line with another study comparing these three groups [20]. However, Monpellier et al. [20] observed least depressive symptoms among patients with no desire for BCS. The tendency for immediate improvement in psychosocial well-being after surgery with a subsequent rebound in psychosocial problems [2, 3] was supported, albeit depended on group belonging. All groups’ body satisfaction improved from baseline to 5 years, in line with some longitudinal studies [18, 34]. However, both groups without BCS had decreased body satisfaction from one to 5 years, while participants with BCS did not and reported a continued increase in satisfaction from baseline to 5 years (Fig. 1). Mental health improved only in the BCS group from baseline to 5 years. Participants desiring BCS had a decline in anxiety and depressive symptoms from baseline to 1-year post-surgery but experienced a subsequent increase (Fig. 2). Their mean levels of symptoms indicated that some individuals probably struggled with anxiety and depressive issues both before and after surgery. Overall, analyses of changes over time support previously observed benefits of BCS [1, 6].

Undergoing BCS requires sufficient weight loss and stability. A commonly used limit for eligibility in Norway is *BMI* < 28 kg/m^2^ [36]. Many subjects desiring BCS did not meet this requirement, which may explain why they had not undergone BCS. One-year post-surgery 76% in the D group had a BMI above 28, and at 5 years, the part was 85%. Research suggest that a higher %TWL is associated with improved mental health [20, 23, 25] and body satisfaction [23, 24], which corresponds well with the BCS group in the present study. Less weight loss could lead to more depressive symptoms and body dissatisfaction and thus be primary to group allocation in explaining variation along these variables. This was accounted for by controlling for BMI/%TWL when analyzing group differences. BMI/%TWL was associated with body satisfaction at all time-points, but only with mental health at 5 years. Participants with no desire for BCS had the lowest %TWL after surgery but did not differ from BCS participants on body satisfaction, suggesting that the relevance of %TWL for body satisfaction vary across groups and individuals. Although the D group had the highest BMI at baseline, and less weight loss compared to the BCS-group, group differences in body satisfaction and mental health, both before and after bariatric surgery, persisted after adjusting for BMI/%TWL. In this study, observed group differences in body satisfaction and mental health must thus be because of something more than mere weight differences.

As previous studies including similar groups have been cross-sectional with post-surgery data, the most novel findings were group differences observed at baseline. The D group displayed the highest levels of body dissatisfaction and scored adversely on all mental health variables relative to the BCS group. Although this study did not examine causality, research has suggested that such factors are associated. Low resilience levels indicated that they could be less well equipped to cope with psychological problems compared to patients who subsequently underwent BCS. High levels of depressive symptoms have, in turn, been linked to body dissatisfaction in patients with obesity [37, 38]. Low levels of resilience among those desiring BCS were primarily found for appraisal of social resources and family cohesion. Support from clinicians and health services might be especially important for this group. The study implies that screening for resilience, body satisfaction, and mental health prior to surgery could be relevant in identifying subjects that are less likely to benefit from surgery, to undergo BCS, and that might need extra attention and guidance. Future research could include within-group analyses of the relationship between resilience, body image, and mental health to further enlighten on why some patients seem to benefit more from surgery. Study strengths include the prospective design, validated instruments, and a relatively large number of participants compared to existing longitudinal studies. However, the sample size was not optimal for within-group comparisons. The low number of patients with no desire for BCS may have resulted in an underestimation of group differences.

## Conclusions

A large proportion of post-bariatric surgery patients desire but do not undergo BCS. This group seemed to struggle more with body image and mental health both before and after bariatric surgery compared to the other groups. Within-group changes over time suggest that post-surgery disparities in body image and mental health are not merely a result of differences in baseline characteristics but may in part be associated with the beneficial effects of BCS.
